# Chlorogenic Acid as a Promising Tool for Mitigating Chilling Injury: Cold Tolerance and the Ripening Effect on Tomato Fruit (*Solanum lycopersicum* L.)

**DOI:** 10.3390/plants13152055

**Published:** 2024-07-25

**Authors:** Mihaela Iasmina Madalina Ilea, Pedro Javier Zapata, Christian Fernández-Picazo, Huertas María Díaz-Mula, Salvador Castillo, Fabián Guillén

**Affiliations:** Agro-Food and Agro-Environmental Research and Innovation Center (CIAGRO-UMH), Postharvest Research Group of Fruit and Vegetables, University Miguel Hernández, Ctra. Beniel km. 3.2, 03312 Orihuela, Spain

**Keywords:** *Solanum lycopersicum* L., storability, ripening, cold tolerance, polyphenol

## Abstract

Tomato fruit (*Solanum lycopersicum* L.) has a very brief storability, displaying chilling injury (CI) when stored in cold conditions used to delay ripening. For this reason, in this study, different concentrations (10, 50, and 100 mg L^−1^) of chlorogenic acid (ChA) were assayed to evaluate its effectiveness in maintaining fruit quality traits and mitigating CI symptoms in tomatoes. Our results showed that ChA treatments effectively delayed weight loss and maintained fruit firmness, with optimal results observed at 50 mg L^−1^. In general, higher concentrations did not result in significant quality improvements. Additionally, ChA-treated tomatoes exhibited reduced values in malondialdehyde (MDA) content and electrolyte leakage (EL), indicating improved membrane integrity and reduced oxidative damage. ChA treatments also maintained a higher total phenolic content (TPC) during storage, with significant levels of individual polyphenols such as rutin, neochlorogenic acid, and p-coumaric acid, suggesting enhanced antioxidant capacity and better preservation of fruit quality. This is the first time the potential of ChA to reduce CI has been evaluated in any fruit species, and its impact in tomato ripening is shown to uphold fruit quality during cold storage, prolonging the storability of tomatoes. In particular, we highlight its natural origin and effectiveness as a postharvest treatment.

## 1. Introduction

Tomato (*Solanum lycopersicum* L.) is a commonly consumed fruit, highly valued for its nutritional benefits (including vitamins, carotenoids, and phenolic compounds) and with economic relevance for growers [1]. However, its postharvest storability is notably brief, presenting a significant challenge for both the agricultural industry and consumers. This limited shelf life is largely attributed to the rapid processes of ripening and senescence that accelerate after harvesting. For this reason, in general, storage and transportation of fruit and vegetables occur under refrigeration to delay metabolism and ripening. However, cold storage (below 10–12 °C) leads to tomato chilling injury (CI), resulting in discoloration, surface softening, and shrinkage [2,3]. This physiopathy promotes fungal infections, resulting in decay. Different studies have proposed increasing tomato storability by controlling ethylene production using 1-methylcyclopropene (1-MCP) [4,5], polyamines [6], and nitric oxide [7]. Physical technologies such as postharvest LED lighting [8] and irradiation [9] or physical barriers such as plastic films for modified-atmosphere packaging [10], active packaging [11], or edible coatings [12] are also able to control ethylene and tomato fruit ripening. On the other hand, plant-origin substances such as plant hormones [13,14] and elicitors [15,16] have been employed to increase the tomato shelf life. Plant-based treatments have increased the interest in this regard. These treatments are sustainable, safe for consumption, and offer additional benefits such as enhancing the antioxidant properties. Among these natural compounds, polyphenols are particularly notable for their crucial roles in plant secondary metabolism, involvement in defense mechanisms, and maintenance of the cellular antioxidant status [17].

Additionally, polyphenols have antioxidant properties that benefit both human health and the quality of the fruit during storage. Among polyphenols, flavonoids like quercetin, naringenin, and rutin, and chlorogenic acid (ChA) as a phenolic acid compound, are the most important phenolic compounds found in tomatoes [18]. ChA is one of the most extensively studied polyphenols due to its diverse biological functions. This phenolic acid is widely distributed in numerous fruits and vegetables, and in tomatoes, its content varies significantly during ripening and senescence processes. Research has demonstrated that the ChA content in tomatoes decreases markedly as the fruit matures [19]. Initially, tomatoes exhibit appreciable levels of this compound, but these levels decline as postharvest ripening progresses. This reduction can affect the antioxidant capacity of the fruit, which in turn influences its firmness and resistance to diseases during storage. Specifically, ChA has shown antimicrobial and antioxidant activities, suggesting its potential for use as a postharvest treatment to extend the tomato shelf life [20]. Additionally, some polyphenols, including ChA and rutin, have been associated with the stimulation of growth regulators such as auxins [21].

Despite the potential benefits of ChA, its direct application to extend storability has not been extensively studied. Previous studies have evaluated the use of polyphenol extracts and chlorogenic acid in different fruits, showing promising results in reducing senescence and improving postharvest quality [22,23,24,25,26]. However, ChA has not been tested in tomatoes for this specific purpose, and to date, no research has evaluated exogenous ChA in relation to cold tolerance in vegetable products. Therefore, a natural plant substance, ChA, was studied to determine its potential in enhancing resistance to CI and reducing senescence in tomatoes, which formed the main objectives of the present study. 

## 2. Results and Discussion

### 2.1. Chlorogenic Acid Impact on Weight Loss Fruit Softening and Chilling Injury Incidence

**Figure 1:**
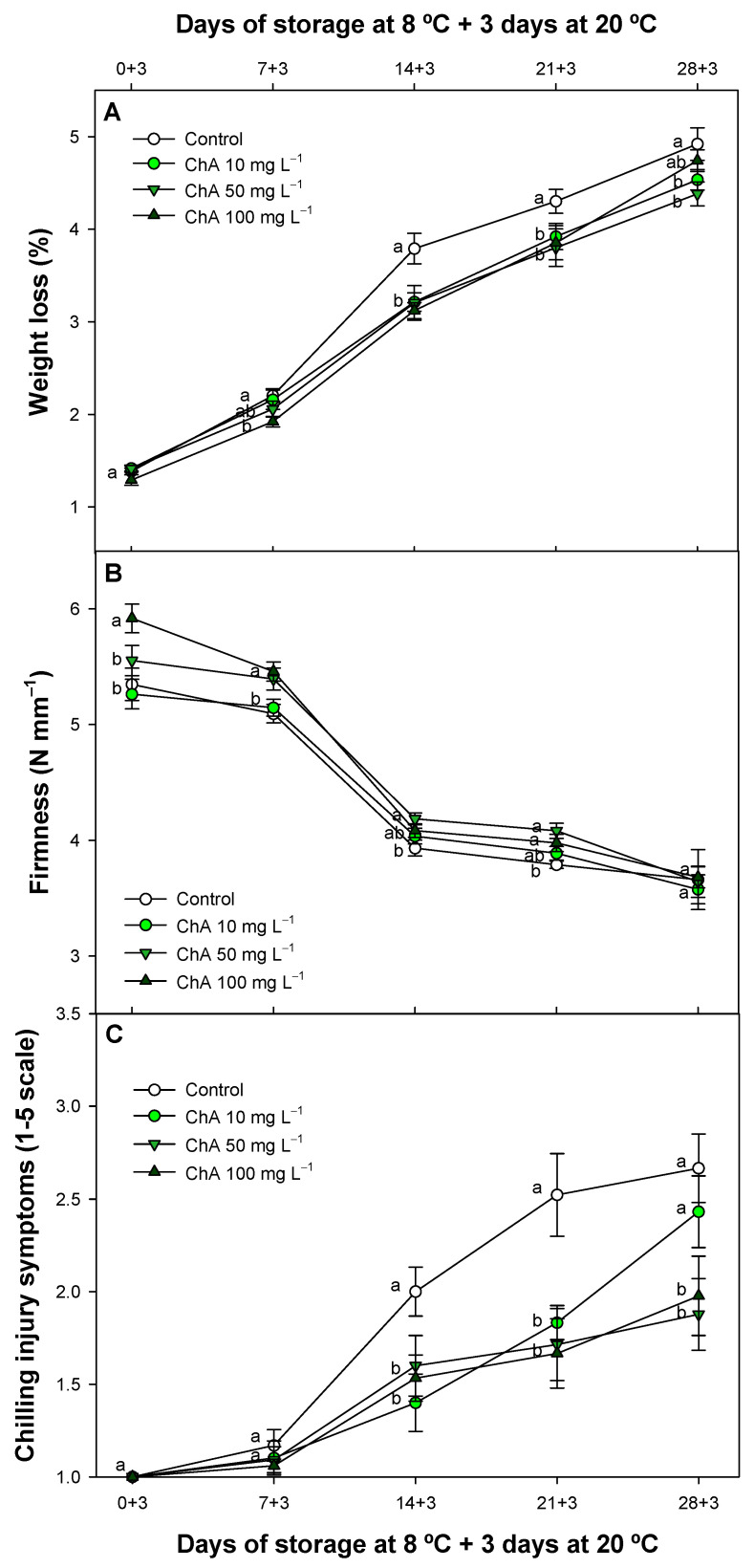
Weight loss (%) (**A**), fruit firmness (N mm^−1^) (**B**), and chilling injury symptoms (**C**) of control tomato (*Solanum lycopersicum* L.) fruit and treated with different chlorogenic acid concentrations and maintained at 8 °C and subsequently for 3 days at 20 °C. Different lowercase letters denote significant differences (*p* < 0.05) among treatments on the same sampling day. Data are the mean ± SE (*n* = 3).

Tomato weight loss arises during the refrigerated period and subsequent shelf life, as expected across all evaluated fruit batches (Figure 1A). However, we observed a significant (*p* < 0.05) ChA impact, reducing the progression of this parameter, with ChA-treated tomatoes (21 + 3) showing similar values to control batches (14 + 3). Despite this delay, no dose-dependent effect was observed, as all ChA-treated batches displayed a similar pattern of weight loss evolution. Specifically, after being refrigerated for 2 weeks and then kept at 20 °C for 3 days, the fruit batches treated with ChA showed reduced weight loss as compared to untreated control batches. Similarly, this trend was observed in fruit firmness, where ChA-treated fruit exhibited a delayed evolution as compared to control fruit values throughout the study (Figure 1B). At 0 + 3 days into the experiment, there was a notable dose-dependent effect, although this effect diminished over the course of the experiment among the highest concentrations applied. ChA at 10 mg L^−1^ displayed the lowest impact, considering the average values obtained between the ChA-treated batches. On the other hand, ChA did not completely inhibit CI (Figure 1C), but it did significantly (*p* < 0.05) mitigate this disorder by around 30–40% after cold storage throughout this study.

Fruit weight loss in tomatoes and other fresh produce can result in shriveling after a water loss of between 3 and 5% [27]. However, ChA was effective in delaying this parameter, maintaining visual properties for a longer duration. Additionally, positive effects were observed in fruit firmness throughout the storage period, as fruit with lower water loss remained firmer. Weight loss is primarily due to the transpiration process, which can be enhanced by higher metabolism and a reduction in the integrity of the plant tissues, making the fruit softer [28]. In this sense, ChA postharvest treatments have demonstrated a positive impact on delaying fruit weight loss and maintaining fruit firmness. For example, ChA applied as an edible coating delayed fruit softening and weight loss in a dose-dependent manner in apricots [29] and lychee [25]. In lychee, 50 mg L^−1^ was identified as the optimal concentration, similar to apricots, while 100 mg L^−1^ was proposed as the ideal concentration to maintain fruit firmness and reduce weight loss in apples [30]. Our results, in agreement with other authors [24], suggest that chlorogenic acid could maintain cellular integrity in tissues, thereby reducing transpiration. This effect is mainly due to the effect of ChA regulating senescence-related enzyme activities and increasing the antioxidant balance in plant cells, thus preventing oxidation and the disassembly of membrane tissues [30,31]. As far as we know, ChA has never been evaluated as a postharvest technology to increase cold tolerance during storage in any fruit species. However, different authors have applied different polyphenol extracts or substances [22,25] to reduce the browning impact in lychee fruit and in pummelo fruit [26] since polyphenols can reduce oxidation processes. In fact, new composite coatings on cherry tomatoes have been linked to a higher polyphenol content, which correlates with a reduced CI impact, lower weight loss, and slower fruit softening evolution [32,33]. Accordingly, it is reasonable to assume that these factors could explain why CI in tomato fruit was delayed during storage. In this context, in potatoes, a higher polyphenol content of ChA and caffeic acid has been associated with CI protection [34]. This is consistent with our results in ChA-treated fruit; for this fruit, the effects on different parameters related to CI, such as MDA and EL, are explained below.

### 2.2. Effect of Chlorogenic Acid on Malondialdehyde Content and Electrolyte Leakage

**Figure 2:**
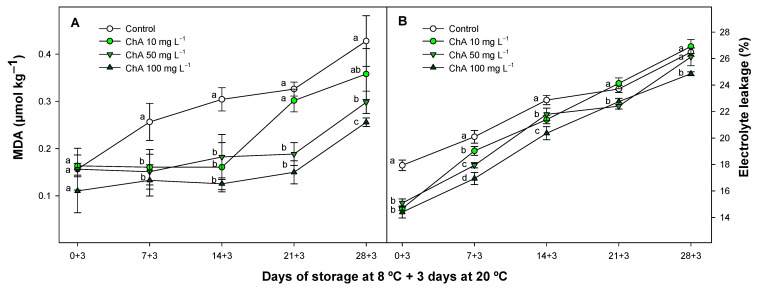
Malondialdehyde (MDA) content (μmol kg^−1^) (**A**) and electrolyte leakage (EL) (%) (**B**), in control tomato (*Solanum lycopersicum* L.) fruit or treated with chlorogenic acid at different concentrations, maintained at 8 °C and subsequently for 3 days at 20 °C. Different lowercase letters denote significant differences (*p* < 0.05) among treatments on the same sampling day. Data are the mean ± SE (*n* = 3).

MDA is a major product obtained after lipid peroxidation, standing as a crucial marker of metabolic breakdown. An increase in this aldehyde has been directly associated with increased membrane permeability [35] and directly related to CI intensity, as previously reported in climacteric fruits such as tomato [36] and in non-climacteric fruits, increasing EL [37,38]. In this sense, management is necessary with different postharvest technologies such as calcium, elicitors, or 1-MCP. Tomato fruit treated with ChA showed a delayed evolution as compared to untreated batches at day 7 during cold storage plus an additional period of 3 days at 20 °C (Figure 2A).

It is important to highlight that the effectiveness of ChA on MDA levels exhibited a dose-dependent manner, especially after two weeks of cold storage. The positive effect of the various ChA concentrations applied was evident, with lower MDA levels associated with higher cell membrane integrity, making the cells less susceptible to damage by reactive oxygen species (ROS). Similarly, Fan et al. [39] found that applying ChA to pitaya fruit helps to maintain the quality of fresh-cut red pitaya. This effect was linked to the preservation of the antioxidant activity levels, reducing oxidation. On the other hand, a reduction in unsaturated fatty acids can influence the alteration of membrane lipids, reducing cold tolerance in different fruit species [40,41]. In this sense, changes in the lipid composition during membrane damage led to decompartmentalization and EL [42]. Regarding EL, a dose-dependent effect between the ChA concentration applied and the positive effect of reducing EL was generally observed throughout the entire experiment (Figure 2B). Control fruit showed increased ion efflux, displaying significantly higher values compared to ChA-treated fruit, especially during the first two weeks of storage. The reduced EL in ChA-treated fruit could explain the positive effect on CI observed previously, as several authors have confirmed in different studies [43,44] in which the lower EL was related to a higher level of polyphenol content, in consensus with our study, as we will describe in Section 2.5.

### 2.3. Effect of Chlorogenic Acid on Respiration and Ethylene Production

**Figure 3:**
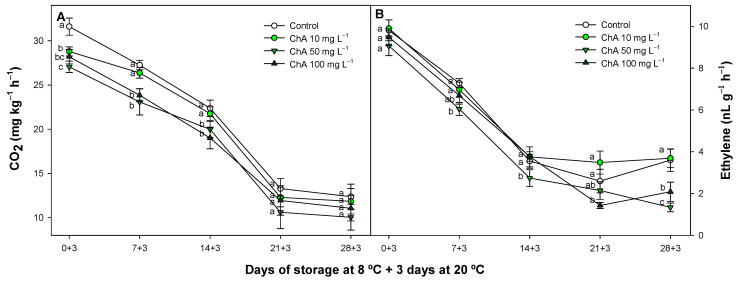
Respiration rate (mg CO_2_ kg^−1^ h^−1^) (**A**) and ethylene production (nL g^−1^ h^−1^) (**B**), in control tomato (*Solanum lycopersicum* L.) fruit or treated with chlorogenic acid at different concentrations, maintained at 8 °C and subsequently for 3 days at 20 °C. Different lowercase letters denote significant differences (*p* < 0.05) among treatments on the same sampling day. Data are the mean ± SE (*n* = 3).

After refrigerated storage, the respiration and ethylene production of tomatoes exhibited a declining trend throughout the experiment for all treated batches evaluated (Figure 3). However, different results (*p* < 0.05) were obtained between the higher concentrations in ChA-treated groups and the control fruit, with the latter generally showing higher average values for these metabolic parameters.

The most concentrated ChA solutions applied resulted in lower average values for both respiration and ethylene production. As ripening progresses, oxidative damage may increase due to enhanced respiration, coinciding with a decrease in the internal level of ChA in tomato fruit [19]. This relationship could explain the reduced respiration observed in ChA-treated fruit. Additionally, the lower oxidative damage might be associated with the previously described lower MDA content and electrolyte leakage percentage. Furthermore, reduced respiration and ethylene production are also linked to decreased weight loss and maintained fruit firmness. Ethylene reduction is directly related to delayed ripening or senescence and higher CI tolerance in climacteric [45] or non-climacteric fruits [46], aligning with our global results. A lower ethylene production is related with a lower metabolism and catabolic reactions. Xi et al. [47] elucidated in apples that the enzyme catalyzing the conversion of malate to pyruvate and NADPH (substrates for respiration in tomatoes and other fruits) was inhibited by 50 mg L^−1^ of ChA. This inhibition could explain the suppressed general metabolism observed after ChA applications, as Shu et al. [30] also confirmed the increased energy balance in cells in ChA-treated apples. In this sense, applying novel technologies focused on reducing tomato CI such as hydrogen sulfide or methyl jasmonate as postharvest applications has shown that increasing the energy balance is crucial to control this disorder [48,49]

### 2.4. Effect of Chlorogenic Acid on Fruit Color Parameters

**Figure 4:**
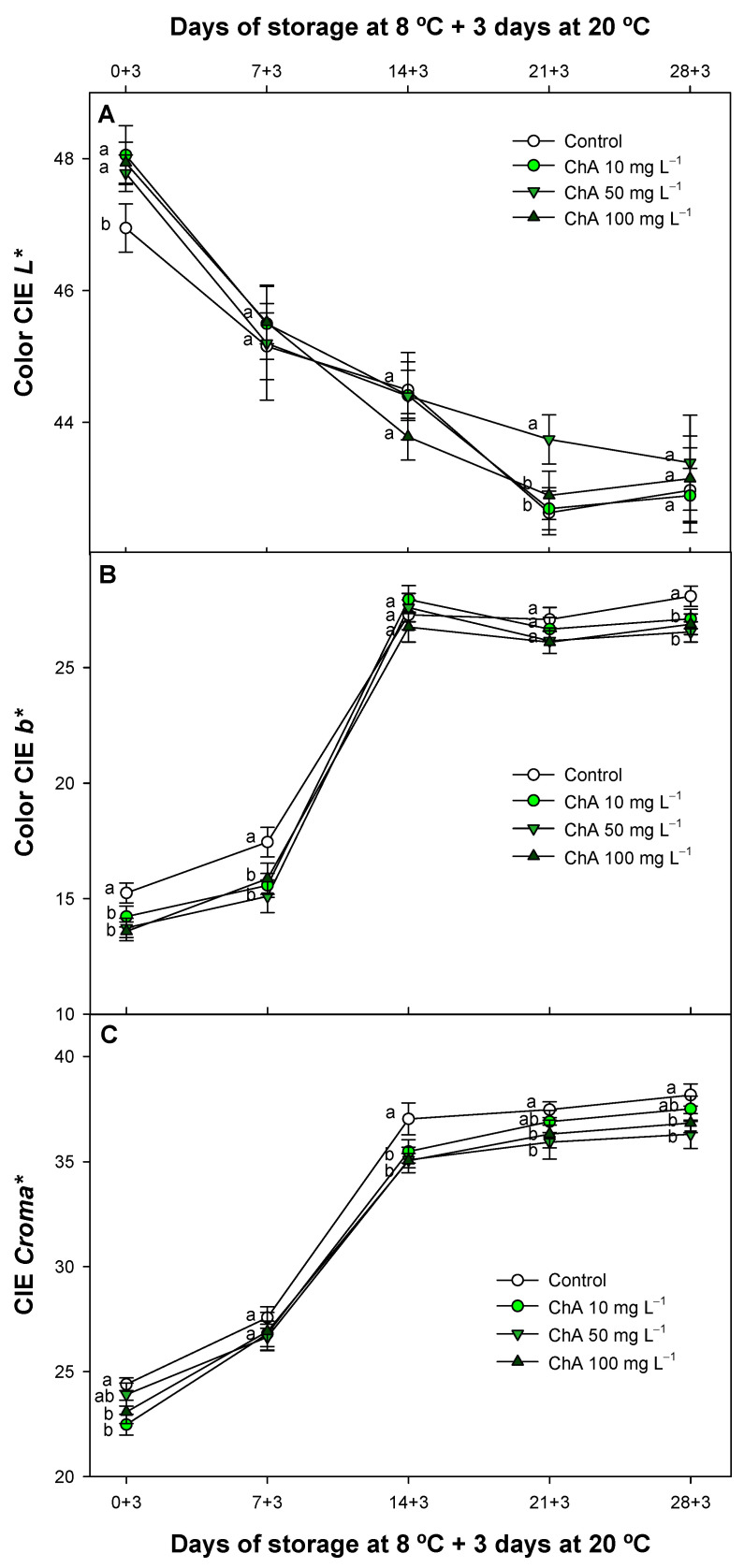
External color evolution CIE L* (**A**), CIE b* (**B**), and CIE Chroma* (**C**) evaluated in control tomatoes (*Solanum lycopersicum* L.) and treated with chlorogenic acid at different concentrations, maintained at 8 °C and subsequently for 3 days at 20 °C. Different lowercase letters denote significant differences (*p* < 0.05) among treatments on the same sampling day. Data are the mean ± SE (*n* = 3).

Tomato color is a crucial factor affecting both market standards and consumer acceptance, making its maintenance a primary objective for producers and distributors. During commercial postharvest, maintaining color is essential as it directly influences perceived quality and demand. As in the present study, applying several new strategies based on the antioxidant potential of different plant regulators and edible coatings has shown that reducing CI during storage is widely related to the ripening process, thus delaying color development [50,51,52]. In this study, several color parameters were obtained, although only those affected by the ChA treatments are described. ChA treatment of tomatoes significantly (*p* < 0.05) affected all parameters displayed in Figure 4. Regarding tomato fruit lightness (CIE L*), all ChA concentrations delayed this parameter after storing the fruit for 3 days at room temperature (Figure 4A). However, these differences disappeared over the course of the experiment, and only the 50 mg L^−1^ concentration maintained higher L* values after 21 days of refrigerated storage plus 3 days at 20 °C. Additionally, the evolution of CIE b* was also delayed during storage in relation with control batches, especially at the beginning of the experiment. All ChA concentrations exhibited a similar delayed pattern, but no important differences were displayed (Figure 4B). ChA concentrations significantly delayed (*p* < 0.05) the intensity and color vividness (CIE Chroma*), particularly at the highest concentrations throughout the entire experiment. These results can be related to a delayed pattern of ethylene production, as previously described for ChA-treated fruit [5]. Given that ethylene detection is necessary for the ongoing ripening of tomato fruit, the slower progression of CIE L* values was associated with a reduced weight loss pattern and a delayed postharvest ripening by Nunes and Emond [53]. These authors described how decreased water evaporation in tomatoes helps to maintain their freshness for extended periods. Tomatoes are rich in polyphenols but with a lower concentration than carotenoids and mainly constitute flavonoids and phenolic acids, with ChA being one of the predominant polyphenols [54]. Polyphenols are mainly accumulated in the tomato skin, contributing not only to the nutritional value and health benefits but also to tomatoes’ sensory qualities, including color stability thanks to antioxidant molecules during ripening [54,55]. In this sense, ChA applied to nectarines as a postharvest treatment delayed all color parameters studied at 25 and 50 mg L^−1^, with a more pronounced effect observed at the higher concentration [56]. This observation aligns with the outcomes of our research.

### 2.5. Effect of Chlorogenic Acid on Phenolic Compounds

**Figure 5:**
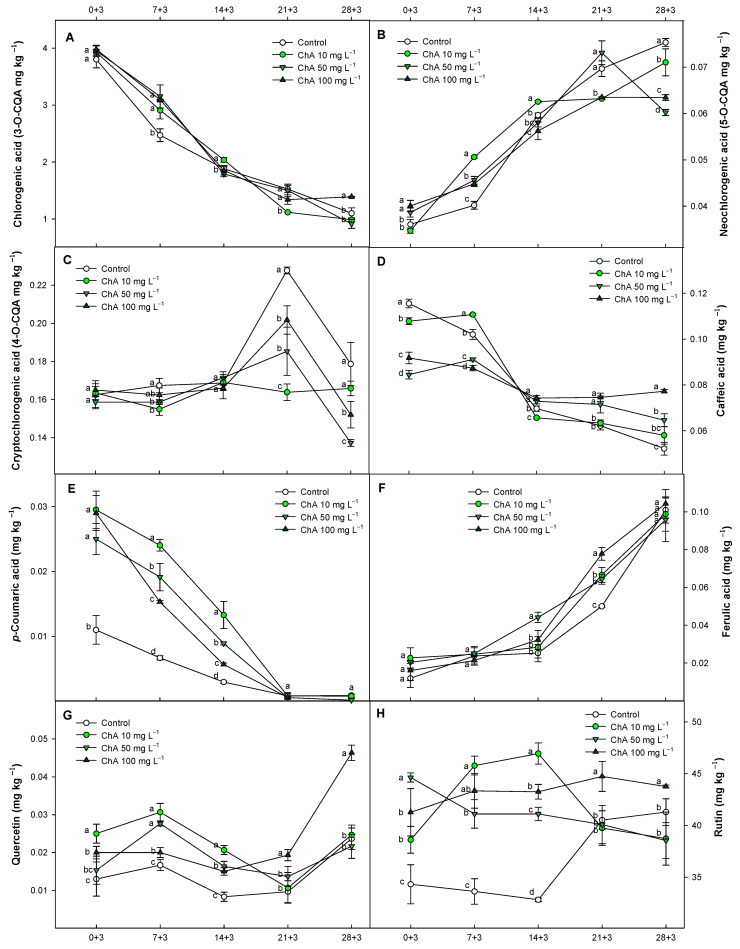
Content of individual phenolic compounds (mg kg^−1^) for chlorogenic acid (**A**), neochlorogenic acid (**B**), cryptochlorogenic acid (**C**), caffeic acid (**D**), *p*-coumaric acid (**E**), ferulic acid (**F**), quercetin (**G**) and rutin (**H**), evaluated in control tomatoes (*Solanum lycopersicum* L.) and treated with chlorogenic acid at different concentrations, maintained at 8 °C and subsequently for 3 days at 20 °C. Different lowercase letters denote significant differences (*p* < 0.05) among treatments on the same sampling day. Data are the mean ± SE (*n* = 3).

In tomato fruit, the most significant phenolic compounds are flavonoids and phenolic acids. Different phenolic compounds were detected in this study (Figure S1 and Table S1). Among the individual phenolic acids, in general, the isomers of caffeoylquinic acid (CQA) were the most prevalent (Figure 5A–C). 

The primary phenolic acid in ‘Kabrera’ tomato fruit was ChA (3-O-CQA) (Figure 5A), and in lower concentration, we detected 4-O-CQA (cryptochlorogenic acid) (Figure 5C) followed by 5-O-CQA (neochlorogenic acid) (Figure 5B). These three isomers were previously detected in tomatoes by Slimestad and Verheul [57]. They were also observed in different cultivars of tomato fruit [58]. Other phenolic acids identified, in descending order of concentration, included caffeic acid and ferulic acid (Figure 5D,F) at similar levels, with p-coumaric acid (Figure 5E) having the lowest concentration. Regarding flavonoids, rutin (Figure 5G) displayed the highest concentration, followed by quercetin (Figure 5F), which had one of the lowest concentrations among all phenolic compounds evaluated in this tomato cultivar. The application of ChA in tomato fruit significantly (*p* < 0.05) enhanced overall levels in phenolic compounds throughout this experiment. However, 4-O-CQA (cryptochlorogenic acid) and caffeic acid (Figure 5C,D) exhibited a delayed accumulation pattern during storage. On the other hand, the application of immersion treatments based on ChA showed no significant (*p* > 0.05) rise in ChA levels within tomato tissues after 3 days at 20 °C. We hypothesize that ChA was immediately metabolized after application, likely affecting the accumulation observed in 5-O-CQA and the other phenolic compounds evaluated over the same storage period.

Recent studies by several authors have reported similar concentrations of the phenolic compounds evaluated in this study, such as ChA, 5-O-CQA, or 4-O-CQA in tomato fruit [59,60,61]. Additionally, the levels of additional phenolic acids, such as p-coumaric, ferulic, and caffeic acids [54,61,62], along with flavonoids such as quercetin and rutin [54,63], were consistent with those reported in previous studies on tomato fruit. In addition, ChA levels have been observed to decline throughout the ripening process, consistent with our results [58,62]. However, while other authors observed a decreased pattern in ferulic acid in the hybrid ‘BHN-589’ during tomato ripening [62], our results showed an increase in this phenolic acid over the storage time. In general, the pattern of increase or decrease during ripening of the different phenolic compounds was similar to those found by other authors for caffeic, p-coumaric, and ferulic acids and for the flavonoid evolution during tomato ripening [58]. The ChA effect in increasing the different phenolic compounds could lead to a potent effect in enhancing the antioxidant defense system in fruits by stimulating the expression of antioxidant enzymes, as has been described in different fruit species for polyphenol applications [17,47,64]. The levels of distinct phenolic compounds in tomatoes can be influenced by extraction techniques, the cultivar evaluated, the ripening stage, and methodological evaluation. These factors can explain the varying phenolic contents reported by different authors [60,61]. Palumbo et al. [65] did not observe a clear pattern in phenolic acids such as those studied in this research exerted by suboptimal temperatures in nectarine, so it seems the different effects displayed by treatments in terms of phenolic acid concentration are solely due to the treatments applied. In this sense, a low-oxygen atmosphere reduced CI in apples by delaying the evolution of ChA and 5-O-CQA, thereby reducing the accumulation of these metabolites and their subsequent oxidation [66]. Postharvest applications of 24-epibrassinolide in guava fruit increased the flavonoid content under suboptimal temperatures, and this increase was related to a lower CI [67]. This delay in phenolic acid catabolism and the increased flavonoid content were also observed in our results.

### 2.6. Effect of Chlorogenic Acid on Total Polyphenol Content Total Soluble Solids and Titratable Acidity throughout Storage

**Figure 6:**
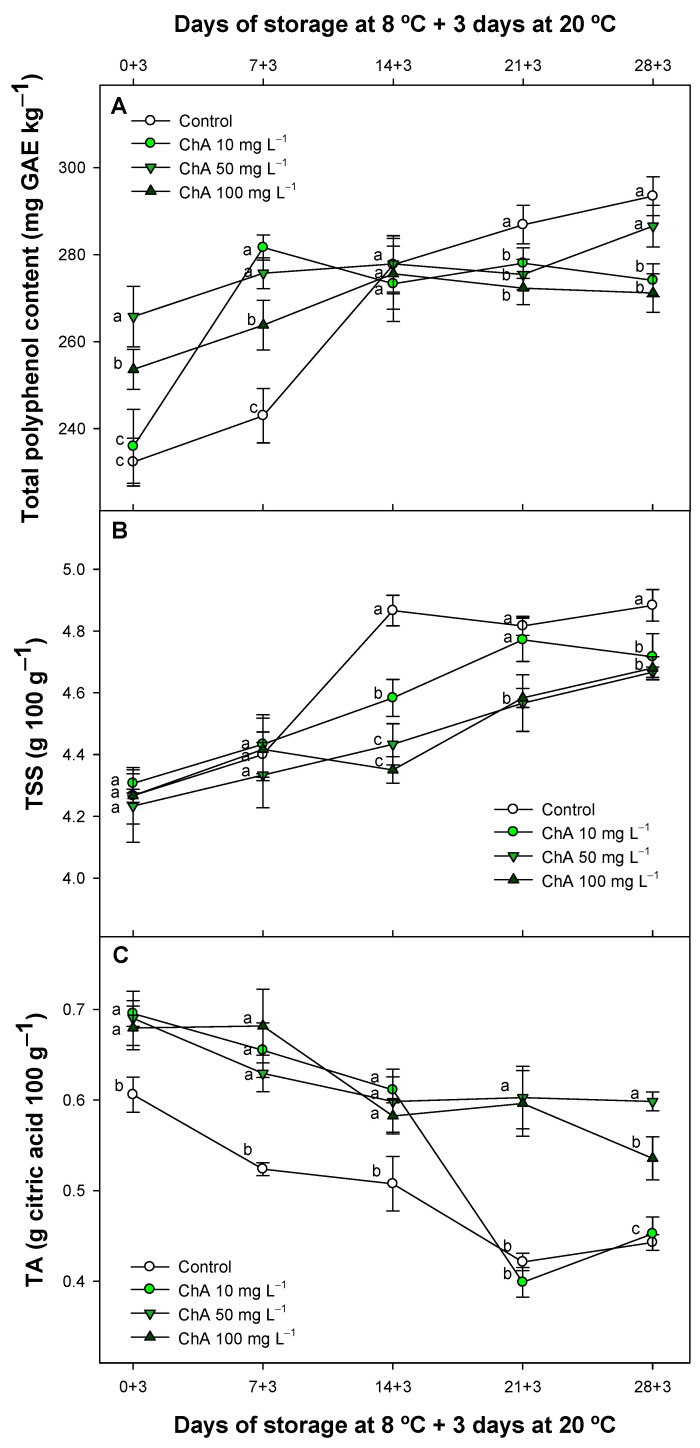
Total polyphenol content (mg GAE kg^−1^) (**A**), total soluble solids (TSS) (g 100 g^−1^) (**B**), and titratable acidity (TA) (g 100 g^−1^) (**C**) of control tomato (*Solanum lycopersicum* L.) and ChA-treated batches maintained at 8 °C and subsequently for 3 days at 20 °C. Different lowercase letters denote significant differences (*p* < 0.05) among treatments for each sampling day. Data are the mean ± SE (*n* = 3).

TPC levels were significantly different (*p* < 0.05) between treated batches only at the beginning of the experiment, with higher TPC levels in ChA-treated fruit (Figure 6A). 

TPC in each sample increased, reaching levels comparable to findings reported by other researchers for tomatoes [68,69,70]. Initially, control fruit batches exhibited a delayed pattern in TPC, but at the conclusion of this study, both control batches and treated fruit with ChA at 50 mg L^−1^ reached the highest TPC values (293.44 ± 4.50 and 274.09 ± 3.81 mg GAE kg^−1^, respectively), with no significant differences found between them (*p* > 0.05). Moreover, TPC levels in all ChA-treated samples were lower as compared to control fruit reaching the final storage period.

In accordance with our results, TPC increased during ripening in tomatoes, as observed by different authors. [19,68]. In line with our findings, although the level of antioxidant substances such as ChA and caffeic acid decreased during the experiment, rutin remained quite stable, with higher values throughout the entire period in ChA-treated fruit. However, other highly antioxidant polyphenolic compounds, such as 5-O-CQA, 4-O-CQA, or p-coumaric acid, showed greater levels in ChA-treated tomatoes only in the initial period of the experiment, with these differences disappearing or showing reduced levels relative to control batches in the final period of storage. These varying patterns may be due to the various functions of specific polyphenols in plants. In tomato fruit, a higher level of TPC has been related to reduced CI after postharvest treatments such as LED lighting [8], UV irradiation [70], hot water treatments [71], and applications with elicitors [67,72]. Moreover, a higher TPC content indicates a higher level of ripening in tomato fruit, suggesting a delayed ripening pattern in ChA-treated fruit likely due to lower respiration and metabolism, as previously described in this study. On this matter, when total soluble solids (TSS) and titratable acidity (TA), which are metabolism substrates, increased and decreased, respectively, during the experiment, ChA postharvest treatments delayed TSS accumulation and total organic acid catabolism significantly (*p* < 0.05). This effect is consistent with delayed ripening, primarily due to lower respiration and ethylene production, as observed in previous tomato studies [4,73], and these metabolic changes happen during the normal ripening process of tomatoes, as previously documented [74]. ChA could contribute to an optimal energy balance in cells, as has been proven for apples [30], reducing excessive breakdown of substrates [23]. In this sense, higher levels of TSS and TA observed in various fruit species after different postharvest treatments have been associated with lower incidences of CI. This effect could be related to the sugars and organic acid contents (such as ascorbic and citric acid), since a cryoprotectant impact of these substances has been observed at increased concentrations [75,76,77].

## 3. Materials and Methods

### 3.1. Plant Material and Experimental Design

Tomatoes (*Solanum lycopersicum* L.) were hand-picked from a commercial orchard in Aguilas (Murcia, Spain) in a green matured color. On the same day, 300 tomatoes without visual damage or disease were brought to the laboratory, where they were sorted for a consistent size. Following this process, the tomatoes were divided into groups per treatment, with 5 fruits per replicate (*n* = 3) for each sampling date. Distilled water immersions (10 min) were used as the control treatment. For ChA treatments, the tomatoes were immersed in 10, 50, and 100 mg L^−1^ solutions for 10 min. In all ChA solutions and control dips, Tween 20 (0.05%) was added. After treatment, tomatoes were allowed to air dry at 20 °C for 1 h prior to being kept at 8 °C and 90% RH for 28 days, followed by an additional period of 3 days at 20 °C for later assessments.

### 3.2. Postharvest Quality Traits

Tomato weight losses were evaluated as a percentage of the initial fruit weight. To measure fruit firmness, tomatoes were tested individually using a TX-XT2i texture analyzer equipped with a flat probe, exerting sufficient pressure to reach 5% fruit deformation in relation to the fruit’s diameter (Stable Microsystems, Godalming, UK). Data obtained have been reported taking into account the ratio of applied force and the distance travelled by the probe (N mm^−1^) and displayed as the mean ± SE. CI in tomatoes was visually evaluated by 9 trained assessors, who scored the surface damage on a scale of 1 to 5: 1 = not affected, 2 = 1–25% surface, 3 = 25–50% surface, 4 = 50–75% surface, and 5 >75% surface. The malondialdehyde (MDA) content in tomato tissue was analyzed following the method previously described [78]. A 2.5 g sample was manually grounded with a mortar and subsequently was mixed using a 10% trichloroacetic acid solution (10 mL). After centrifuging the mixture at 4 °C and 10,000× *g* for 20 min, the supernatant was obtained, and 2 mL was mixed with 6 mL of 0.67% thiobarbituric acid and homogenized. The samples were heated (95 °C for 20 min) and then brought to ambient temperature before their evaluation at different wavelengths (450, 532, and 600 nm) with a spectrophotometer (1900 UV/Vis, Shimadzu, Kyoto, Japan). These evaluation were conducted in duplicate and reported as μmol kg^−1^.

Electrolyte leakage (EL) was measured following a previously described method [79]. From each replicate, 15 disks (1 cm diameter) were obtained from the outer tomato rind with a cork borer, after removing the interior matrix and rinsing. The disks were rinsed 3 times with deionized water for 3 min each, then immersed in deionized water (50 mL) for 1 h at room temperature and in continuous agitation. Initial electrical conductivity (C1) at 20 °C was recorded. They were subsequently heated (121 °C for 15 min) and tempered to ambient temperature, and conductivity (C2) was recorded. EL results were obtained with this formula: (C1/C2) × 100. The respiration rate was determined by taking 5 fruits from each replicate and treatment, and placing them inside hermetically sealed 3.7 L containers for 60 min. A gas sample (1 mL) was taken from the headspace in sextuplicate, then CO_2_ and ethylene production were measured in a Shimadzu 14B and a Shimadzu GC 2010, respectively (Shimadzu Europa GmbH, Duisburg, Germany), following the method reported previously [80]. For CO_2_ evaluation, the instrument contained a catarometric detector and a 3 m stainless steel column with an inner diameter of 3.3 mm filled with chromosorb 102. The column was maintained at a temperature of 55 °C, and the injector and detector were set at 110 °C. For ethylene production determination, the instrument was equipped with a flame-ionization detector and a 3 m stainless steel column (inner diameter of 3.5 mm) packed with activated alumina of 80/100 mesh. The column was kept at 70 °C, with the injector and detector maintained at 110 °C. The results obtained were displayed as mg of CO_2_ kg^−1^ h^−1^, and nL g^−1^ h^−1^ for ethylene concentration. External tomato color was evaluated at three different surface points around the equator of 5 fruits per replicate with a CRC 400 colorimeter (Minolta Camera Co., Tokyo, Japan). After halving the tomatoes, around 50 g from one half of each fruit in each replicate was squeezed through two layers of cotton cloth, and the total soluble solids (TSS) content was evaluated in duplicate for each replicate using a refractometer Atago PR-101 at 20 °C (Atago Co., Ltd., Tokyo, Japan). In the same way, juice titratable acidity (TA) was also measured in duplicate using 1 mL of diluted juice (25 mL) with an automatic titrator (785 DMP Titrino, Metrohm, Herisau, Switzerland). TSS and TA were expressed as g per 100 g^−1^ and g of citric acid equivalents per 100 g^−1^, respectively.

### 3.3. Polyphenolic Content

To determine the polyphenol content, the remaining half of each tomato was pulverized using liquid nitrogen. Then, 2 g of the frozen tomato powder was homogenized in 10 mL of a methanol (8:2) solution containing 2 mM sodium fluoride to control polyphenol oxidase activity and avoid polyphenol breakdown. The mixture was then subjected to centrifugation (10,000× *g* at 4 °C, 15 min). Supernatants were reacted in duplicate with a Folin–Ciocalteu solution to evaluate the total phenolic content, as has been previously reported for plant tissues [81]. Two 200 μL aliquots (for duplicate analysis) of one extract per replicate (*n* = 3) were taken for analysis. Each aliquot was combined with 300 μL of 50 mM phosphate buffer solution, 2.5 mL of Folin–Ciocalteu reagent, and 2 mL of 1 N Na_2_CO_3_. The mixture was shaken and then incubated in a water bath at 50 °C for 5 min. A blank sample was prepared by substituting the extract with the previously described methanol solution. Absorbance was measured at 760 nm using a UV-1700 spectrophotometer (Shimadzu). Results were expressed in mg of gallic acid equivalents (fresh weight) based on a gallic acid calibration curve. 

For the evaluation of individual polyphenols, one sample per replicate was obtained by extraction from 5 g of pulverized tomato in 5 mL of methanol, homogenizing for 1 min (Ultraturrax, T18 basic, IKA, Berlin, Germany). After centrifugation at 10,000× *g* and 4 °C for 15 min, samples were filtered and analyzed using an LC-MS/MS 8050 (Shimadzu, Japan). The conditions applied are described as follows: a nebulizer flow rate and a drying gas flow rate of 3 L min^−1^ and 10 L min^−1^, respectively; 250 °C was the desolvation line temperature and 400 °C was the heat block temperature of the selected ion monitoring; −35 V was the collision energy value; and we employed a full MS scan mode ranging from 100 to 1000 *m*/*z*. A Mediterranea SEA18 column (10 mm length × 0.21 mm internal diameter, 2.2 μm particle size, Teknokroma, Barcelona, Spain) was used for the chromatographic separation at 40 °C. A 0.1% formic acid solution for phase A and 0.1% formic acid in acetonitrile (ACN) for phase B were used with deionized water for the mobile phase. The gradient elution programmed was 0–2 min with 5% B, 2–10 min with 95% B, 10–11 min with 95% B, 11–12 min with 5% B, and 12–16 min with 5% B, with a flow rate of 0.400 mL min^−1^ and using an injection volume of 10 µL. External standards facilitated the quantification of phenolic compounds. Instrument control and data analysis were performed with the Labsolutions LCMS software version 5.98 (Shimadzu). All analyses were performed in triplicate.

### 3.4. Statistical Analysis

Data obtained were the mean ± SE, and those were analyzed using analysis of variance (ANOVA) tests. Significant differences (*p* < 0.05) among means were identified using Tukey’s HSD test. Treatments that differed significantly within the same sampling period were indicated by different lowercase letters. All statistical analyses were performed using the SPSS software package, version 22 (IBM Corp., Armonk, NY, USA).

## 4. Conclusions

ChA demonstrates significant potential as a new postharvest technology to maintain tomato quality, thus increasing cold storability. Our study has showed that ChA treatments effectively delay weight losses and fruit softening. This effect was particularly pronounced at higher concentrations of ChA, but the optimal results were obtained at 50 mg L⁻¹, considering that higher concentrations did not result in a significant quality increase. Additionally, ChA-treated tomatoes exhibited lower levels of malondialdehyde and electrolyte leakage, indicating improved membrane integrity and reduced oxidative damage. These results confirmed and explained the positive impact of ChA in reducing chilling injury symptoms. Furthermore, the role of ChA in maintaining a higher TPC during storage suggests an enhanced antioxidant capacity, contributing to better preservation of the fruit quality. This is the first evidence of the potential of ChA to reduce chilling injury in any fruit species, highlighting ChA as a sustainable and effective postharvest treatment to control disorders during cold storage and prolong the storability of tomatoes. However, further research into its applications across various fruit species is needed.

## Data Availability

The original contributions presented in this study are included in the article. Further inquiries can be directed to the corresponding author.

## Supplementary Materials

The following supporting information can be downloaded at: https://www.mdpi.com/article/10.3390/plants13152055/s1, Figure S1: LC-MS/MS Chromatogram view MRM of the phenolic acids and flavonoids detected in tomato samples at different retention times (RT); Table S1: Phenolic compounds content (mg kg^−1^) evaluated in control tomatoes and treated with chlorogenic acid at different concentration maintained at 8 °C and 3 days at 20 °C.
