# Supplementary material for: Chlorogenic Acid as a Promising Tool for Mitigating Chilling Injury: Cold Tolerance and the Ripening Effect on Tomato Fruit (Solanum lycopersicum L.)
Source: Plants (Basel). 2024 Jul 25;13(15):2055. doi: 10.3390/plants13152055 (PMC11314013; doi:10.3390/plants13152055)
Supplement: Supplementary file 1 [file plants-13-02055-s001.zip › plants-3097418-supplementary.pdf]

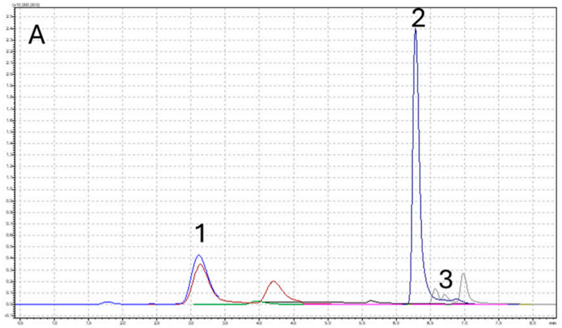

Figure A:Chromatogram View MRM

1: Chlorogenic acid; RT: 3.121

2: Rutin; RT: 6.300

3: Cryptochlorogenic; RT: 6.762

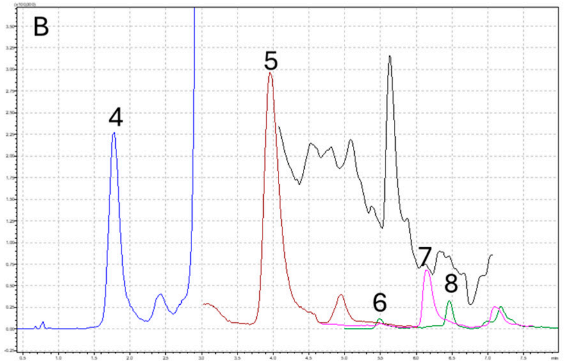

Figure B: Chromatogram View MRM

4: Neochlorogenic acid; RT: 1.756

5: Caffeic acid; RT: 3.942

6: *p*-coumaric acid; RT: 5.576

7: Ferulic acid; RT: 6.161

8 : Quercetin; RT: 6.494

Figure S1. LC-MS/MS Chromatogram view MRM of the phenolic acids and flavonoids detected in tomato (*Solanum lycopersicum* L.) samples at different retention times (RT).

Table S1. Phenolic compounds content (mg kg^{-1}) evaluated in control tomatoes (*Solanum lycopersicum* L.) and treated with chlorogenic acid at different concentration maintained at 8 °C and 3 days at 20 °C. Different lowercase letters denote significant differences ($p < 0.05$) among treatments on each sampling day. Data are the mean ± SE (n = 3).

**Phenolic acids (mg kg $^{-1}$) and treatment**	**Storage time (days at 8 ºC + 3 days at 20 ºC)**				
Chlorogenic acid	**0+3**	**7+3**	**14+3**	**21+3**	**28+3**
Control	3.80 ± 0.14a	2.47 ± 0.11b	1.88 ± 0.03b	1.52 ± 0.05a	1.10 ± 0.09b
CGA 10 mg L^{-1}	3.92 ± 0.05a	2.90 ± 0.15a	2.03 ± 0.03a	1.11 ± 3.54e^{-3}b	0.98 ± 0.02b
CGA 50 mg L^{-1}	3.95 ± 0.09a	3.15 ± 0.20a	1.79 ± 0.04b	1.50 ± 0.11a	0.91 ± 0.07b
CGA 100 mg L^{-1}	3.97 ± 0.05a	3.09 ± 7.46e^{-3}a	1.85 ± 0.08b	1.34 ± 0.08a	1.38 ± 0.01a
Neochlorogenic acid	**0+3**	**7+3**	**14+3**	**21+3**	**28+3**
Control	0.03 ± 1.08e^{-3}b	0.04 ± 8.38e^{-4}c	0.06 ± 4.91e^{-4}b	0.07 ± 1.68e^{-3}a	0.07 ± 8.51e^{-4}a
CGA 10 mg L^{-1}	0.03 ± 5.50e^{-4}b	0.05 ± 2.51e^{-4}a	0.06 ±1.76e^{-4}a	0.06 ± 1.73e^{-4}b	0.07 ± 2.91e^{-3}b
CGA 50 mg L^{-1}	0.03 ± 9.73e^{-4}a	0.04 ± 8.18e^{-4}b	0.05 ± 9.33e^{-4}bc	0.07 ± 2.55e^{-3}a	0.06 ± 6.65e^{-4}d
CGA 100 mg L^{-1}	0.04 ± 1.18e^{-3}a	0.04 ± 5.29e^{-4}b	0.05 ± 1.89e^{-3}c	0.06 ± 1.01e^{-4}b	0.06 ± 6.38e^{-4}c
Cryptochlorogenic acid	**0+3**	**7+3**	**14+3**	**21+3**	**28+3**
Control	0.16 ± 7.34e^{-3}a	0.16 ± 3.80e^{-3}a	0.16 ± 1.07e^{-3}a	0.22 ± 1.75e^{-3}a	0.17 ± 0.01a
CGA 10 mg L^{-1}	0.16 ± 3.43e^{-3}a	0.15 ± 3.16e^{-3}b	0.17 ± 4.25e^{-3}a	0.16 ± 4.35e^{-3}c	0.16 ± 3.80e^{-3}a
CGA 50 mg L^{-1}	0.15 ± 2.81e^{-3}a	0.15 ± 1.33e^{-3}b	0.17 ± 3.20e^{-3}a	0.18 ± 0.01b	0.13 ± 1.79e^{-3}c
CGA 100 mg L^{-1}	0.16 ± 3.22e^{-3}a	0.16 ± 3.74e^{-3}ab	0.16 ± 5.55e^{-3}a	0.20 ± 7.45e^{-3}b	0.15 ± 6.89e^{-3}b
Caffeic acid	**0+3**	**7+3**	**14+3**	**21+3**	**28+3**
Control	0.11 ± 1.88e^{-3}a	0.10 ± 2.18e^{-3}b	0.07 ± 1.22e^{-3}b	0.06 ± 1.83e^{-3}b	0.05 ± 2.72e^{-3}c
CGA 10 mg L^{-1}	0.10 ± 1.49e^{-3}b	0.11 ± 4.04e^{-4}a	0.06 ± 5.29e^{-4}c	0.06 ± 1.34e^{-3}b	0.05 ± 3.86e^{-3}bc
CGA 50 mg L^{-1}	0.08 ± 1.89e^{-3}d	0.09 ± 6.56e^{-4}c	0.07 ± 1.15e^{-3}a	0.07 ± 3.74e^{-3}a	0.06 ± 2.83e^{-3}b
CGA 100 mg L^{-1}	0.09 ± 2.52e^{-3}c	0.08 ± 1.13e^{-3}d	0.07 ± 1.19e^{-3}a	0.07 ± 1.82e^{-3}a	0.07 ± 7.51e^{-4}a
p-Coumaric acid	**0+3**	**7+3**	**14+3**	**21+3**	**28+3**
Control	0.01 ± 2.19e^{-3}b	6.70e^{-3} ± 3.21e^{-4}d	2.96e^{-3} ± 1.85e^{-4}d	8.56e^{-4} ± 3.52e^{-4}a	9.01e^{-4} ± 2.53e^{-4}a

CGA 10 mg L^{-1}	0.03 ± $2.89e^{-3}$a	0.02 ± $8.95e^{-4}$a	0.01 ± $2.09e^{-3}$a	$8.36e^{-4}$ ± $5.26e^{-4}$a	$8.00e^{-4}$ ± $3.52e^{-4}$a
CGA 50 mg L^{-1}	0.02 ± $2.39e^{-3}$a	0.01 ± $2.10e^{-3}$b	$8.91e^{-3}$ ± $2.31e^{-4}$b	$5.78e^{-4}$ ± $1.85e^{-4}$a	$6.80e^{-4}$ ± $3.21e^{-4}$a
CGA 100 mg L^{-1}	0.03 ± $2.69e^{-3}$a	0.01 ± $2.08e^{-4}$c	$5.66e^{-3}$ ±$2.31e^{-4}$c	$6.30e^{-4}$ ±$1.05e^{-4}$a	$6.30e^{-4}$ ±$1.05e^{-4}$a
Ferulic acid	**0+3**	**7+3**	**14+3**	**21+3**	**28+3**
Control	0.01 ± $4.88e^{-3}$a	0.02 ± $4.30e^{-3}$a	0.02 ± $4.60e^{-3}$b	0.05 ± $6.12e^{-4}$c	0.10 ± $6.87e^{-3}$a
CGA 10 mg L^{-1}	0.02 ± $5.35e^{-3}$a	0.02 ± $3.98e^{-3}$a	0.02 ± $5.67e^{-3}$b	0.06 ± $3.98e^{-3}$b	0.09 ± $9.08e^{-3}$a
CGA 50 mg L^{-1}	0.02 ± $5.36e^{-4}$a	0.02 ± $9.71e^{-4}$a	0.04 ± $2.74e^{-3}$a	0.06 ± $2.63e^{-3}$b	0.09 ± 0.01a
CGA 100 mg L^{-1}	0.01 ± $5.33e^{-3}$a	0.02 ± $2.48e^{-3}$a	0.03 ± $5.05e^{-3}$b	0.07 ± $3.35e^{-3}$a	0.10 ± $7.54e^{-3}$a
**Flavonoids (mg kg^{-1}) and treatment**	**Storage time (days at 8 ºC + 3 days at 20 ºC)**				
Quercetin	**0+3**	**7+3**	**14+3**	**21+3**	**28+3**
Control	0.01 ± $4.50e^{-3}$c	0.01 ± $1.45e^{-3}$c	$8.33e^{-3}$ ± $1.20e^{-3}$c	$9.66e^{-3}$ ± $2.90e^{-3}$b	0.02 ± $2.84e^{-3}$b
CGA 10 mg L^{-1}	0.02 ± $2.51e^{-3}$a	0.03 ± $2.33e^{-3}$b	0.02 ± $1.20e^{-3}$a	0.01 ± $4.05e^{-3}$b	0.02 ± $2.60e^{-3}$b
CGA 50 mg L^{-1}	0.01 ± $3.71e^{-3}$bc	0.02 ± $6.66e^{-4}$a	0.01 ± $1.33e^{-3}$b	0.01 ± $2.66e^{-3}$b	0.02 ± $3.18e^{-3}$b
CGA 100 mg L^{-1}	0.02 ± $1.64e^{-3}$b	0.02 ± $1.33e^{-3}$a	0.01 ± $1.00e^{-3}$b	0.01 ± $1.48e^{-3}$a	0.04 ± $2.02e^{-3}$a
Rutin	**0+3**	**7+3**	**14+3**	**21+3**	**28+3**
Control	34.32 ± 1.89c	33.62 ± 1.23c	32.82 ± 0.16d	40.51 ± 0.91b	41.28 ± 1.29b
CGA 10 mg L^{-1}	38.62 ± 1.29b	45.78 ± 0.91a	46.94 ± 1.02a	39.74 ± 1.67b	38.73 ± 2.56b
CGA 50 mg L^{-1}	44.63 ± 0.43a	41.11 ± 1.37b	41.10 ± 0.64c	40.08 ± 1.84b	38.54 ± 1.72b
CGA 100 mg L^{-1}	41.27 ± 2.28b	43.34 ± 1.66ab	43.25 ± 0.71b	44.73 ± 1.44a	43.75 ± 0.14a
